# Differences in PI-RADS Classification of Prostate Cancer Based on mpMRI Scans Taken 6 Weeks Apart

**DOI:** 10.3390/tomography11080092

**Published:** 2025-08-18

**Authors:** Justine Schoch, Viola Düring, Michael Wiedmann, Daniel Overhoff, Daniel Dillinger, Stephan Waldeck, Hans-Ulrich Schmelz, Tim Nestler

**Affiliations:** 1 Department of Urology, Federal Armed Forces Hospital Koblenz, Ruebenacher Strasse 170, 56072 Koblenz, Germany; 2 Department of Diagnostic and Interventional Radiology, Federal Armed Forces Hospital Koblenz, Ruebenacher Strasse 170, 56072 Koblenz, Germany

**Keywords:** MeSH, prostate cancer, mpMRI, MRI-guided biopsy, csPCa, PI-RADS

## Abstract

Objectives: This study aimed to investigate the consistency of lesion identification by Prostate Imaging Reporting and Data System (PI-RADS) and the related clinical and histological characteristics in a high-volume tertiary care center. Materials and methods: The analysis used real-world data from 111 patients between 2018 and 2022. Each patient underwent two multiparametric magnetic resonance imaging (MRI) scans of the prostate at different institutions with a median interval of 42 days between the scans, followed by an MRI-fused biopsy conducted 7 days after the second MRI. Results: The PI-RADS classifications assigned to the index lesions in the in-house prostate MRI were as follows: PI-RADS V, 33.3% (n = 37); PI-RADS IV, 49.5% (n = 55); PI-RADS III, 12.6% (n = 14); and PI-RADS II, 4.5% (n = 5). Cancer detection rates for randomized and/or targeted biopsies were 91.9% (n = 34) for PI-RADS V, 65.5% (n = 36) for PI-RADS IV, 21.4% (n = 3) for PI-RADS III, and 20% (n = 1) for PI-RADS II. Overall, malignant histology was observed in 64.9% (n = 72) of the targeted lesions and 57.7% (n = 64) of the randomized biopsies. In the first performed, external MRI, 18% (n = 20) and 10.8% (n = 12) of the patients were classified in the higher and lower PI-RADS categories, respectively. The biopsy plan was adjusted for 57 patients (51.4%); nevertheless, any cancer could have possibly been identified regardless of the adjustments. Conclusion: The 6-week interval between the MRI scans did not affect the quality of the biopsy results significantly.

## 1. Introduction

Prostate cancer (PCa) is the most frequent malignancy in men [1,2]. Localized PCa is usually asymptomatic, and according to current guideline recommendations, screening and timely detection of significant PCa can reduce mortality due to PCa while preserving the quality of life [3]. Men with more than 15 years of life expectancy should undergo an individual risk-adapted screening approach, including prostate-specific antigen (PSA) test and digital rectal examination (DRE), after being counseled on the potential risks and benefits [4]. After an initial suspicion of PCa, a systematic transrectal ultrasound (US)-guided biopsy is typically offered to the patients.

Alternatively, multi-parametric magnetic resonance imaging (mpMRI) of the prostate can play a crucial role in diagnostic evaluations aiming to reduce unnecessary systematic biopsies and improve the detection of clinically significant PCa [5,6]. To standardize the interpretation and reporting of prostate lesions from mpMRI, the Prostate Imaging Reporting and Data System (PI-RADS) classification was established by the PI-RADS Steering Committee [7]. In the early phases of MRI-guided prostate biopsies, an additional in-house prostate MRI following an external MRI was often necessary to mark the lesions for biopsy.

The current study aimed to examine the repeatability of lesion identification using mpMRI and the associated clinical and histological characteristics in a high-volume tertiary care center that performs approximately 500 biopsies annually.

## 2. Materials and Methods

### 2.1. Patients

This study utilized real-world data from 111 patients with suspected PCa in the Bundeswehrzentralkrankenhaus Koblenz between 2018 and 2022. During this period, a second in-house MRI was necessary for lesion marking prior to performing a fusion-guided prostate biopsy. After 2022, this additional scan was no longer required. From the initial cohort of patients referred for prostate biopsy between 2018 and 2022, we included only those who presented with an external mpMRI indicating a suspicious lesion and who subsequently underwent a second in-house mpMRI prior to fusion biopsy. Patients were excluded if external radiology reports were missing or if histopathological data were incomplete.

Indications for requiring MRI included suspicious PSA, abnormal DRE findings, and abnormal transrectal US findings.

### 2.2. mpMRI

Each patient underwent two mpMRI scans of the prostate at different institutions, with a median interval of 42 days (interquartile range [IQR] 31.75–52.25) between the scans. Radiologists rated and reported the MRI results according to the PI-RADS version 2.1 [7]. All readings were performed independently and blinded to the results of the previous MRI to reduce potential bias. Although the specific scanner types and manufacturers from external institutions were not consistently documented due to the retrospective and multicenter nature of the study, all examinations met the minimum technical requirements specified by PI-RADS v2.1 and were deemed diagnostically sufficient by the evaluating institution. The in-house MRIs were interpreted by a board-certified uroradiologist with at least five years of experience.

### 2.3. Biopsy

Men with PI-RADS lesions graded ≥3 underwent MRI-targeted and systematic biopsies, whereas those with PI-RADS 2 underwent only systematic biopsies. Biopsy was performed at a median of 7 days (IQR 4–10) after the second MRI by an experienced urologist who typically performs >50 biopsies annually. A predefined software-assisted template was used for institutional standardized biopsy in all patients. After rectal cleansing, biopsies were performed under antibiotic prophylaxis. A software-assisted fusion technique (BioJet Systems rev. 3.0) was used. All the cores were documented and histopathologically evaluated. Clinically significant PCa (csPCa) was defined as any PCa with a Gleason score of 3 + 4 or higher (International Society of Urological Pathology [ISUP] Grade Group ≥ 2), and insignificant PCa (insigPCa) was defined as that with a Gleason score of 3 + 3 (ISUP 1). The number of positive cores and the ISUP grades for any detected cancer were recorded.

### 2.4. Statistics

Statistical analyses were performed using IBM SPSS Statistics for Windows (v 24.0) (Armonk, NY, USA). Categorical variables are presented as n (%). Medians and IQRs were reported for continuous variables. Data were analyzed using Pearson’s chi-square test. For each PI-RADS group, the 95% confidence intervals of the csPCa detection rates were calculated using the exact Clopper–Pearson method. Multivariate binary logistic regression analysis was performed to evaluate potential predictors of clinically significant prostate cancer, including age, PSA level, digital rectal examination findings, prostate volume, PSA density, and internal PI-RADS classification. Differences were considered statistically significant at *p* < 0.05.

## 3. Results

**Figure 1:**
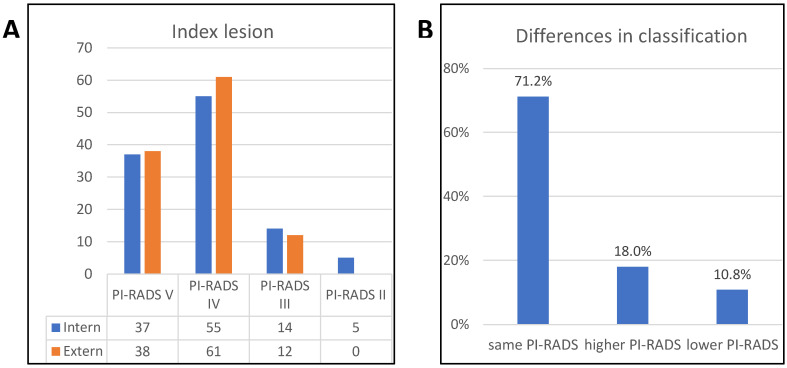
Different PI-RADS classifications in internal and external MRI. Results of internal and external mpMRI of 111 patients were compared (**A**) and found to differ significantly (*p* < 0.001); 71.2% (n = 79) were rated in the same PI-RADS category, 18% (n = 20) had a higher PI-RADS score in the external MRI, and 10.8% (n = 12) had a lower score (**B**).

**Figure 2:**
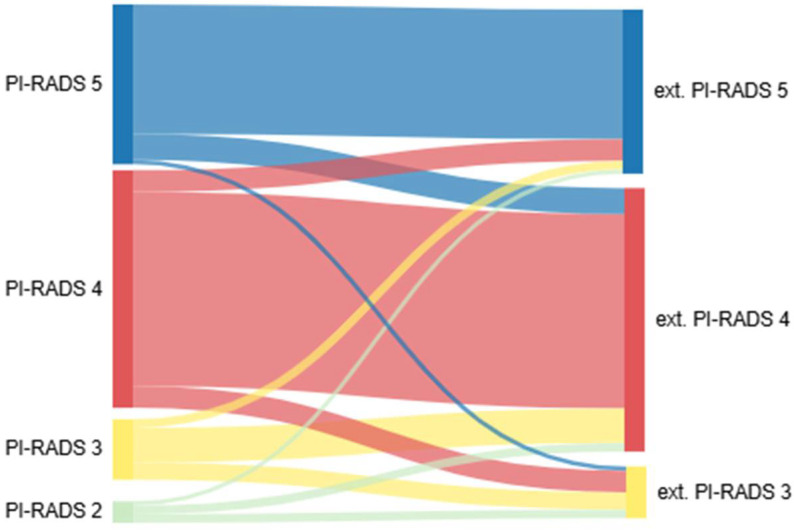
Detailed distribution of different PI-RADS classifications. Sankey plot of differences in PI-RADS classification.

Data from 111 patients treated between 2018 and 2022 were analyzed. The patient characteristics are summarized in Table 1. Patients with at least one suspicious lesion in an externally performed mpMRI of the prostate were referred to our hospital. The highest-scoring PI-RADS classification was defined as the index lesion. For multiple lesions with the highest PI-RADS score, the one with the largest diameter was considered the index lesion. Distributions of index lesions in the in-house mpMRI were PI-RADS V at 34.2% (n = 38), PI-RADS IV at 55% (n = 61), and PI-RADS III at 10.8% (n = 12). We performed a repeated mpMRI with a median interval of 42 days [31.75–52.25] between the scans. The distribution of suspicious index lesions in the in-house mpMRI was as follows: 91.9% (95% CI: 78.1–98.3%) for PI-RADS V, 63.6% (95% CI: 49.5–75.2%) for PI-RADS IV, 21.4% (95% CI: 4.7–50.8%) for PI-RADS III, and 20.0% (95% CI: 0.5–71.6%) for PI-RADS II. (Figure 1A). The PI-RADS classification of the index lesions was similar in 71.2% (n = 79) of the patients with a Cohen’s kappa of 0.508, indicating moderate agreement, while 18% (n = 20) had a higher PI-RADS classification, and 10.8% (n = 12) had a lower PI-RADS classification on external mpMRI (Figure 1B, *p* < 0.001). Detailed differences in the PI-RADS classifications are shown in Table 2 and Figure 2. The majority of patients had only one lesion (67.6%, n = 75), three patients (2.7%) had no detectable lesion (PI-RADS II), and 33 patients (29.7%) had more than one lesion in the mpMRI performed in-house. Most lesions were located in the peripheral zone of the prostate (68.8%, n = 75). The multivariate regression analysis did not reveal any statistically relevant associations between the investigated parameters (age, PSA level, digital rectal examination findings, prostate volume, PSA density, and internal PI-RADS classification) and the presence of clinically significant prostate cancer, and confidence intervals were generally wide, limiting interpretability.

**Figure 3:**
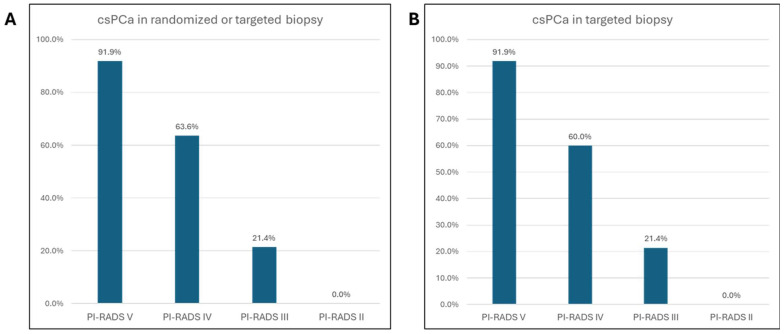
Cancer detection rate within the different PI-RADS classifications. Detection rate of overall csPCa in randomized and/or targeted biopsy (**A**). Detection rate of csPCa in targeted biopsy only (**B**).

After a median of 7 days [4,5,6,7,8,9,10], a targeted and randomized biopsy of the prostate was performed based on internal mpMRI. Most patients underwent biopsy (80.2%, n = 89); 19.8% were previously biopsied (n = 22), and 5.4% were under active surveillance (n = 6) (Table 3). While the cancer detection rate was 69.4% (n = 77), an atypical small acinar proliferation (ASAP) or high-grade prostatic intraepithelial neoplasia (HGPIN) was exclusively detected in 2.7% (n = 3) of patients. Among the 77 patients with cancer, 76 (98.7%) had csPCa, and only one had insignPCa in the PI-RADS IV lesion and systematic cores (1.3%). However, 27.9% (n = 31) of patients had benign histopathological results, with no detectable cancer. Clinically significant cancer was detected in 64.0% of the patients (n = 71) in targeted biopsies and 52.3% of the patients (n = 58) in randomized biopsies. Overall, csPCa detection in randomized and/or targeted biopsies was based on the PI-RADS classification, as follows: 91.9% (n = 34) in PI-RADS V, 63.6% (n = 35) in PI-RADS IV, 21.4% (n = 3) in PI-RADS III, and 20% (n = 1) in PI-RADS II (Figure 3A). Among the targeted biopsies, csPCa was detectable in 91.9% (n = 34) of PI-RADS V index lesions, 60% (n = 33) of PI-RADS IV index lesions, 21.4% (n = 3) of PI-RADS III index lesions, and none of the PI-RADS II lesions (Figure 3B).

The biopsy plan was adjusted for 57 patients (51.4%) based on the in-house mpMRI findings. In most cases, the biopsy plan differed in terms of the number of lesions, although lesions with detectable cancer would still have been included. For example, both MRIs described the lesion as clinically significant prostate cancer; however, one of them additionally identified a potential second lesion, with or without histopathological confirmation of carcinoma.

## 4. Discussion

In this study, we compared the results of two mpMRI scans of the prostate performed at an interval of 6 weeks and correlated them with the histological findings after targeted and randomized prostate biopsies. Of the 111 patients, the PI-RADS index lesions were similar in 71% cases; however, 18% had a higher PI-RADS classification in external MRI, while 11% had a lower. Although findings from small subgroups (e.g., patients downgraded from PI-RADS 5 to 4) should be interpreted with caution due to limited statistical power, possible reasons for these differences are discussed below. First, there could be differences in the quality of the MRI scans.

Although the PI-RADS classification requires certain technical specifications, these cannot ensure high-quality scans [8,9]. To evaluate the image quality in a standardized manner, the Prostate Imaging Quality (PI-QUAL) score was implemented in 2020 [10]. It assigns scores from 1 (poor quality) to 5 (excellent quality) based on adherence to international imaging standards across key sequences, such as T2-weighted imaging, diffusion-weighted imaging, and dynamic contrast-enhanced imaging. Furthermore, less-experienced MRI readers have a lower inter-reader agreement in PI-RADS scoring [11]. The learning curve of a radiologist in a self-directed learning system seems to improve rapidly within the first 40 examinations and then decelerates [12]. Dedicated interactive training can improve the accuracy of cancer diagnosis in the future [13,14]. Few studies have assessed inter-scan variability and PI-RADS repeatability of mpMRI from a more radiological and technical viewpoint. Zhang et al. showed that in a single-scanner same-day mpMRI of the prostate, no significant difference in inter-scan, inter-rater, and inter-sequence variability was found [15]. In another study, Fedorov et al. performed mpMRI of the prostate within 2 weeks and suggested high repeatability of PI-RADS II assessments [16]. These findings aligned with those of a small study conducted in 2016 that showed a reasonably small variation in apparent diffusion coefficient measurements in a single-scanner repeated examination without patient motion [17]. Our results showed less accurate inter-test reproducibility, possibly due to inter-test variability.

The study has some limitations. One major limitation of this study is the lack of blinded re-reading of external MRI scans. Due to the retrospective design and lack of access to image data, comparison was based solely on radiology reports. Furthermore, a formal image quality assessment using metrics such as the PI-QUAL score could not be performed due to the lack of external imaging datasets. Although quality variation may have contributed to differences in PI-RADS classification, this hypothesis remains speculative. In addition, some heterogeneity in scanner technology and acquisition protocols between institutions may have influenced the observed variation, even though all scans fulfilled minimum technical standards. Regarding the initial external mpMRI, we have no information regarding the training of readers. However, we know most of the radiologists of the institutions where the mpMRI scans were performed, and the readings were taken by highly experienced radiologists. A further limitation of our study is that the number of biopsies taken was above the average in similar studies, and the targeted area was excluded from the systematic biopsy [18,19]. Nevertheless, evidence shows that at least five cores per target could maximize the cancer detection rates, and considering that the rate of serious complications in 95% of csPCa is detected in the first three cores [20], there is no clear recommendation for the number of cores per target that should be taken.

Owing to improvements in image processing, a second internal mpMRI is no longer necessary, significantly relieving the healthcare system from an economic and financial perspective concerning the limited availability of MRI resources. The biopsy plan was adjusted in 51.4% of cases based on internal mpMRI findings. While no clinically significant prostate cancers appeared to have been missed, all csPCa lesions were located in regions that would have been sampled regardless of classification differences. Based on real-world data, our findings suggest that prostate mpMRI scans performed within a 6-week interval do not appear to meaningfully impact biopsy planning or the detection of clinically significant prostate cancer, which may hold relevant implications for routine clinical decision-making. This conclusion is based on retrospective assessment and not on formal modeling. It should therefore be interpreted with appropriate caution. We are eagerly awaiting further studies on the implementation of artificial intelligence in image interpretation, which could revolutionize the processing and interpretation of mpMRI of the prostate.

## 5. Conclusions

In most patients, the overall PI-RADS assessments of in-house and out-of-house mpMRI were comparable. In approximately 30% of the cases, up- or down-grading occurred during the second mpMRI performed within 6 weeks. Although the biopsy plan was adjusted slightly in approximately 50% of the patients, histopathological results, depending on the localization of the detected cancer, suggested that any cancer could probably have been identified regardless of these adjustments.

## Figures and Tables

**Table 1:** Characteristics of patients.

Characteristics	*n* = 111	%
Age (yr), median (IQR)	67 [62–72]	
PSA level (ng/mL), median (IQR)	7.6 [5.0–10.1]	
<10	76	68.5
≥10	35	31.5
DRE		
Normal	71	64.0
Abnormal	31	27.9
No information	9	8.1
Prostate volume (mL), median (IQR)	52 [37–67]	
PSAD (ng/mL/mL), median (IQR)	0.14 [0.08–0.20]	
Number of lesions		
0	3	2.7
1	75	67.6
2	28	25.2
3	5	4.5
Index lesion		
PI-RADS V	37	33.3
PI-RADS IV	55	49.5
PI-RADS III	14	12.6
PI-RADS II	5	4.5
Location of index lesion		
PZ	75	68.8
TZ	33	30.3
AFS	1	0.9

PSA, prostate-specific antigen; PSAD, PSA density; DRE, digital rectal examination; PI-RADS, Prostate Imaging Reporting and Data System; PZ, peripheral zone; TZ, transitional zone; AFS, anterior fibromuscular stroma.

**Table 2:** Detailed distribution of different PI-RADS classifications.

		External MRI				
		PI-RADS II	PI-RADS III	PI-RADS IV	PI-RADS V	All
Internal MRI	PI-RADS II	0	40% (2)	40% (2)	20% (1)	5
	PI-RADS III	0	28.6% (4)	57.1% (8)	14.3% (2)	14
	PI-RADS IV	0	9.1% (5)	81.8% (45)	9.1% (5)	55
	PI-RADS V	0	2.7% (1)	16.2% (6)	81.1% (30)	37
	All	0	12	61	38	

**Table 3:** Histopathological characteristics.

Characteristics	*n* = 111	%
No cancer/benign	31	27.9
HGPIN	1	1.3
Cancer detection rate	77	69.4
Grade group ≥ 2 (GS ≥ 3 + 4)		
csPCA	76	98.7
insignPCA	1	1.3
Biopsy history		
Biopsy naive	89	80.2
Previously biopsied	22	19.8
AS	4	3.6
Re-biopsy ASAP	2	1.8
Number of biopsies	23 [21–25]	
Number of biopsies in the lesion	7 [5.5–8.5]	
Number of randomized biopsies	13 [11–15]	
PCA in lesion biopsies	72	64.9
PCA in randomized biopsies	66	59.5

ASAP, atypical small acinar proliferation; HGPIN, high-grade prostatic intraepithelial neoplasia; GS, Gleason score; csPCa, clinically significant prostate cancer; insignPCa, clinically insignificant prostate cancer.

## Data Availability

All data analyzed during this study are included in this article. The data are available from the corresponding author upon reasonable request.
